# Oral administration of *Lactobacillus casei* Shirota improves recovery of hand functions after distal radius fracture among elder patients: a placebo-controlled, double-blind, and randomized trial

**DOI:** 10.1186/s13018-019-1310-y

**Published:** 2019-08-14

**Authors:** Chunhua Zhang, Sujuan Xue, Yong Wang, Dan Yu, Limei Hua, Chunhua Guo, Dawei Wang, Min Lei

**Affiliations:** 1 Department of Emergency Medicine, the Third Hospital of Hebei Medical University, No. 139 Ziqiang Road, Shijiazhuang, 050051 Hebei China; 2 Department of Nutrition and Diet, the Third Hospital of Hebei Medical University, No. 139 Ziqiang Road, Shijiazhuang, 050051 Hebei China; 3 Department of Orthopedics, the Third Hospital of Hebei Medical University, No. 139 Ziqiang Road, Shijiazhuang, 050051 Hebei China; 4 Department of Nutrition, Bethune International Heping Hospital, No. 398 Zhong Shan West Road, Shijiazhuang, 050082 Hebei China; 5 Department of Oral and Maxillofacial Surgery, the Third Hospital of Hebei Medical University, No. 139 Ziqiang Road, Shijiazhuang, 050051 Hebei China

**Keywords:** *Lactobacillus casei* Shirota, Distal radius fracture, Michigan Hand Questionnaire, Hand function, Elder patients

## Abstract

**Background:**

To evaluate the effect of oral *Lactobacillus casei* Shirota (LcS) administration on recovery of hand functions in senior patients diagnosed with an acute distal radius fracture.

**Methods:**

This clinical trial is double-blind and placebo-controlled, in which 293 senior patients with distal radius fracture were initially enrolled. After exclusion, 264 eligible patients were randomly assigned to receive oral placebo or LcS daily for a period of 3 months after the fracture. Treatment outcomes were Michigan Hand Questionnaire (MHQ) score, radial deviation and inclination, and ulnar deviation and variance, all of which were monitored and measured every month.

**Results:**

Throughout the length of this study, MHQ score, radial deviation and inclination, and ulnar deviation and variance of patients on oral LcS displayed a significantly faster improvement in comparison to those receiving placebo, over the 3-month intervention period.

**Conclusion:**

Oral administration of LcS dramatically accelerated hand function recovery in senior patients with distal radius fracture.

## Introduction

Fracture of the distal radius is a most common upper extremity injury [1]. Although the epidemiology of forearm fracture does not display an age-dependent exponential increase like those of the hip or the spine, as a result of age-associated increase in osteoporosis incidences as well as decrease of bone mass acquisition, the senior population is more likely to suffer acute distal radius fractures and prone to slower healing processes [2, 3].

Probiotics, which are live microbial dietary ingredients, have been shown to exert several health benefits [4, 5]. Consumption of probiotics, often as dietary supplement in drinks or capsules, is clinically safe as confirmed in patients with various diseases [6–8], such as children who are severely ill [9] or professional athletes [10]. Use of probiotic is also shown to have curative effects in bone-related diseases. For example, probiotics have been reported to affect the gut-brain-bone axis and exhibit beneficial effects on aging bone, as well as osteoporosis [11–13]. In particular, valyl-prolyl-proline is a bioactive peptide produced from fermentation with *Lactobacillus helveticus* and has been demonstrated to enhance bone formation in vitro [14]. It has been consistently shown in various animal models that probiotic treatment prevents loss of bone mass and increases bone mass density [15–17]. *Lactobacillus casei* Shirota (LcS), a commercially available probiotic, reportedly reduced the inflammatory joint injuries in collagen-elicited arthritis, by regulating pro-inflammatory cytokines such as IL-6, IL-10, and TNF-α [18]. LcS was also reported to inactivate NF-κB and consequently the synthesis of COX-2 [19].

To date, the impacts of probiotic treatments on patients with distal radius fracture have not been reported, except for a recent prospective study by our own group [20]. In that clinical trial, preliminary assessments, including the DASH (disabilities of the arm, shoulder, and hand) score, pain, CRPS (complex regional pain syndrome) score, active range of motion, and grip strength, have demonstrated prospective but promising results on the efficacy of LcS on elder patients suffering from distal radius fracture [20]. With the aim to bring more comprehensive observations on the beneficial effects of LcS, we have conducted this current placebo-controlled, double-blind, and randomized clinical trial of similar setting, using recovery of hand functions, in terms of Michigan Hand Questionnaire (MHQ) score, radial deviation, ulnar deviation, radial inclination, and ulnar variance, as the outcome assessments.

## Methods

### Ethics statement

This clinical trial was conducted during August 2015 and August 2017, with the approval of the Ethics Committee of the Third Hospital of Hebei Medical University and in strict conformity with the guidelines stated in the Declaration of Helsinki. All enrolled patients provided informed, written consent forms and agreed to our policy of data utilization.

### Patients

Two hundred ninety-three patients aged 60 years or older, who were diagnosed with a non-displaced fracture of the distal radius and suitable for conservative treatment, were initially enrolled in the present study. All patients received treatments at the Third Hospital of Hebei Medical University. Exclusion criteria include open or intraarticular displaced fractures; history of wrist fractures on either side; high-energy fractures; bilateral fractures; fractures that involve the shaft of the radius, or ulna other than a simple fracture through the styloid; soft tissue infections at the operative site; patients undergoing chemotherapy or radiotherapy, or any chronic medication with known adverse effects on the skeleton; patients who were mentally or physically compromised; and patients who consumed LcS in any form within 6 months before the enrollment into this study. Based on these criteria, 29 out of 293 patients were excluded.

### Randomization and group design

The remaining 264 eligible patients were assigned to LcS or placebo treatments in a random and even manner, using a permuted block randomization method stratified according to their MHQ scores at admission. Then, every patient was instructed to consume 2 daily servings (100 mL per serving) of either skimmed milk that contains a minimum of 1.2 × 10^10^ colony-forming unit (CFU) LcS or skimmed milk alone as the placebo (both provided by Mengniu Co. Ltd.), with one serving at breakfast and the other at dinner, for 3 months since the day after the fracture. LcS contents in the skimmed milk were verified by the State Food and Drug Administration of China. Every week, all patients receive free supplies of skimmed milk, either one of the two types with coded labels to conceal the content to both the investigators and patients. During the study period, patients were asked not to consume any food supplement or medication containing probiotics, other than those provided by the investigators. All patients were re-visited every month to assess the outcome of the treatment as well as to evaluate their compliance to the aforementioned instructions. Eight patients from the LcS group and 11 patients from the placebo group were excluded because of personal reasons or non-compliance to the study instructions.

### Treatment outcome evaluations

All evaluations were conducted by physicians who are blind to the group assignment, both on the day of the fracture as the baseline and at monthly follow-up visits for 3 months. Michigan Hand Questionnaire (MHQ) was used as the primary outcome [21], which includes 63 questions that fall into 6 domains: overall hand function, daily life activities, esthetics, pain, work performance, and satisfaction of the individual with hand functions (12 questions). The domains of work and daily activities are referring to handicap and disability (22 questions), and those of function and pain are referring to symptoms (15 questions). MHQ score ranges from 0 to 100, in which a lower score indicates a higher degree of disability. Secondary outcomes were defined as radial deviation and inclination, and ulnar deviation and variance, which were measured with a goniometer.

### Statistical analysis

Statistical analysis of the current intention-to-treat trial was carried out with the use of the SPSS software (SPSS Inc., USA). Results were presented as mean ± standard deviation unless otherwise stated. Sample size was estimated using our preliminary data by Cohen’s *d* method [22]. The means of parameters from both groups were divided by standard deviation to calculate the standardized effect size, the largest of which was then adopted by Cohen’s *d* power table to determine minimum group size with 5% statistical significance and 90% power. The normality of data distribution was determined with the Kolmogorov-Smirnov goodness-of-fit test. The Mann-Whitney test was performed to evaluate non-normally distributed data, ANOVA followed by Tukey’s post hoc test was utilized to analyze normally distributed data, and the *P* values < 0.05 indicated statistical significance.

## Results

**Fig. 1:**
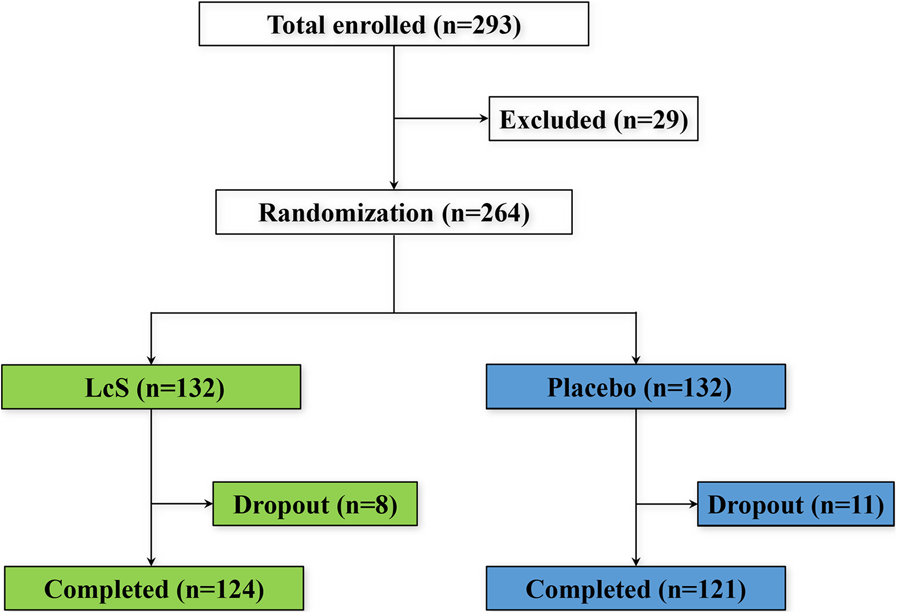
Flow diagram of the study design

The design of the present study is illustrated as a flow diagram in Fig. 1. Two hundred ninety-three patients were enrolled into the present trial, in which 29 were excluded for they failed to meet the inclusion criteria. The remaining 264 eligible patients were assigned into two treatment groups in a random and even fashion. Compared with capsules or other forms, skimmed milk exhibits optimal preservation of probiotic bioactivities and promotes the maximum willingness of the patients to consume, therefore was chosen as the ideal LcS vehicle. All patients were expected to consume 2 daily servings of either skimmed milk that contains a minimum of 1.2 × 10^10^ colony-forming unit (CFU) LcS or identical skimmed milk alone as the placebo, with one serving at breakfast and the other at dinner, for 3 months since the day after the fracture. Eleven patients from the placebo group and 8 patients from the LcS group were excluded because of personal reasons or non-compliance. One hundred twenty-four patients in the LcS group and 121 patients in the placebo group completed the trial in accordance with the protocol. All 264 eligible patient data were analyzed and hereby presented in the current study. No serious adverse events were observed during the period of the study.

First, we evaluated the general characteristics of the patients in the two treatment groups as listed in Table 1. We found no significant baseline difference between the two groups, in terms of age, gender, height, body weight, hand dominance, injured side, or types of the fracture.

**Table 1:** Baseline characteristics of patients

	LcS (*n* = 132)	Placebo (*n* = 132)	*P* value
Gender (male/female)	62/70	69/63	n.s.
Age (years)	64.9 ± 3.3	65.0 ± 3.8	0.48
Height (m)	1.61 ± 0.22	1.59 ± 0.17	0.42
Body weight (kg)	57.9 ± 5.9	58.7 ± 6.0	0.37
Injured side (right/left)	78/54	80/52	n.s.
Injured side (dominant/non-dominant)	76/56	82/50	n.s.
Fracture classification (AO)			
23A3.2	23	21	n.s.
23A3.3	25	23	n.s.
23C2.1	43	44	n.s.
23C2.2	21	24	n.s.
23C3.2	20	20	n.s.

Values are mean ± SD, *n.s. P* > 0.05

**Fig. 2:**
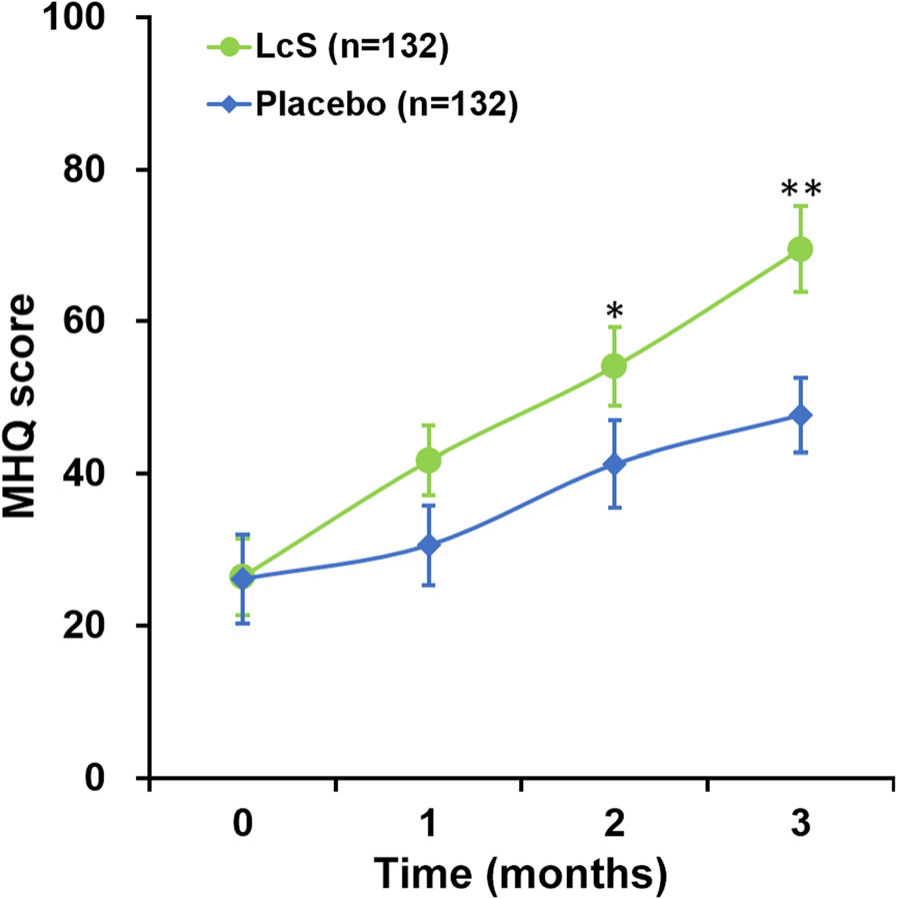
MHQ scores of participants. Values are mean ± SD. *P < 0.05, **P < 0.01, between LcS and placebo at respective time points

All participants were followed up through monthly visits for a 3-month period, to assess the outcome of their treatments. Throughout the study period, a gradual elevation of MHQ scores of all patients was observed, and MHQ scores of patients receiving LcS displayed a markedly faster pace of increase than those of the patients receiving placebo treatment (Fig. 2).

**Fig. 3:**
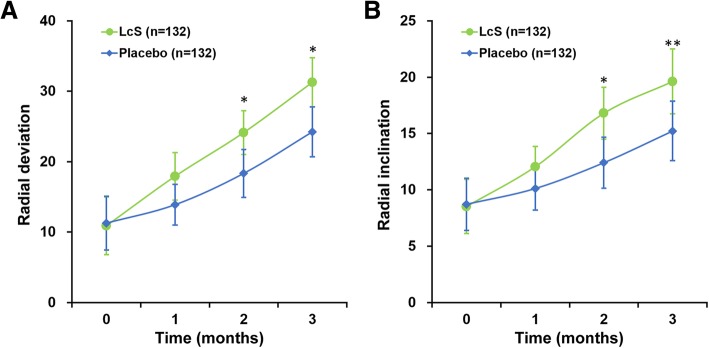
**a** Radial deviation and **b** radial inclination of participants. Values are mean ± SD. *P < 0.05, ** *P* < 0.01, between LcS and placebo at respective time points

**Fig. 4:**
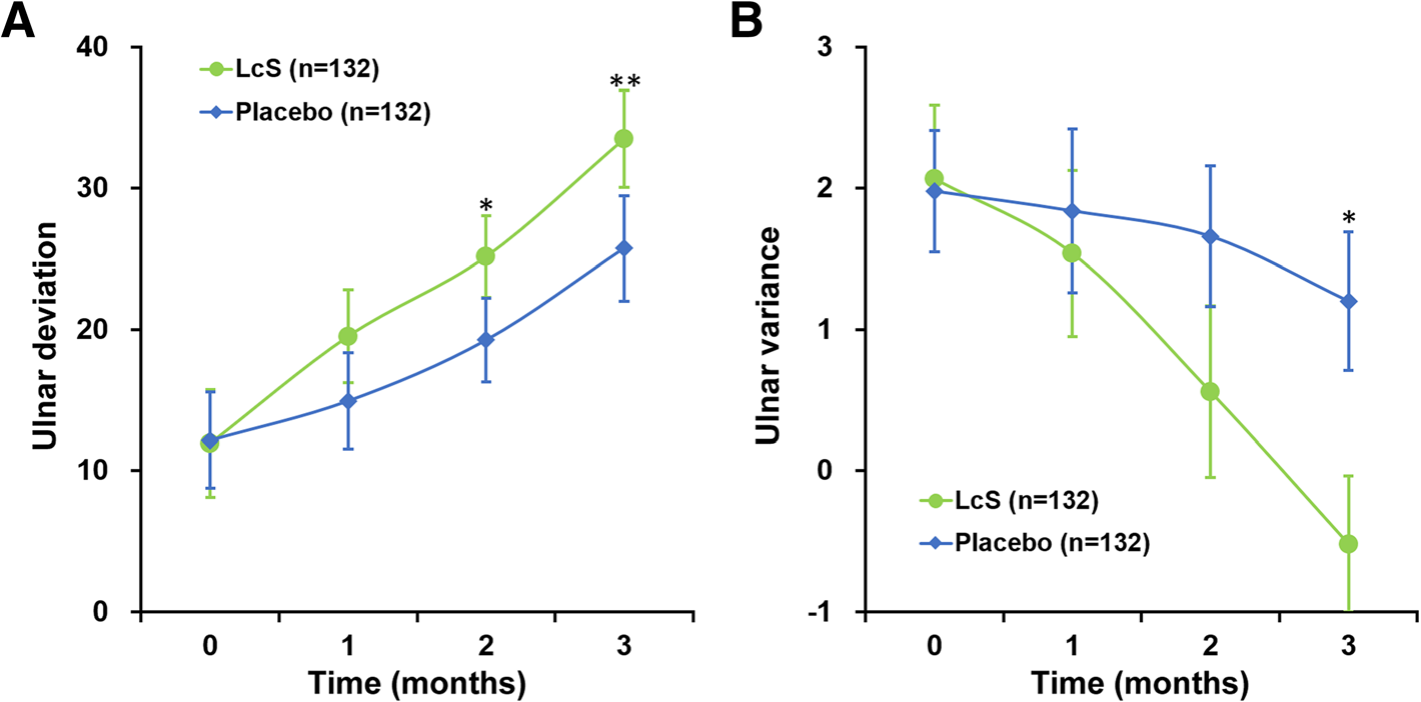
**a** Ulnar deviation and **b** ulnar variance of participants. Values are mean ± SD. *P < 0.05, **P < 0.01, between LcS and placebo at respective time points

As secondary outcomes, we also assessed the radial deviation (Fig. 3a) and inclination (Fig. 3b) of all patients, both of which exhibited a more pronounced increase in LcS group patients than in placebo group patients in a significant manner starting from month 2 till the end of study. Last but not least, two ulnar parameters, namely ulnar deviation and ulnar variance, were also evaluated (Fig. 4). Similar as radial deviation, ulnar deviation in patients administered with LcS displayed a faster trend of elevation than that in patients on placebo over the 3-month study period (Fig. 4a), especially with significant differences in the last two follow-ups (*P* < 0.05). Moreover, ulnar variance showed a steady decline among LcS group patients, whereas a much slower declining pace was observed in the placebo group (Fig. 4b).

## Discussion

In the present clinical trial, we assessed the impacts of the probiotic LcS on distal radius fracture in 264 eligible senior patients. General characteristics of the patients in both treatment groups, including age, gender, height, body weight, hand dominance, injured side, and types of the fracture, were not statistically different. Therefore, the randomization process we used provided comparable baseline endpoints for the rest of the trial.

In our previous study [20], we included DASH (disabilities of the arm, shoulder, and hand) score [23] as one of the primary outcomes. DASH and MHQ scales are utilized for self-evaluation of the upper extremity functions, and both have the major advantage of providing valuable information on the status of the patients when physical measurements are not feasible [24]. However, while both DASH and MHQ specifically measure upper extremity functions, the functional status is assessed through different means. DASH emphasizes on global upper-extremity disability and symptoms, including psychological, physical, and social aspects [25, 26], while MHQ addresses satisfaction and esthetics in a way distinct from DASH [27]. In the present study, primary outcome was assessed using the MHQ score at both baseline and all subsequent monthly re-visits. MHQ scores of all patients gradually increased during the recovery phase after the fracture. However, the elevation of MHQ scores of patients who received LcS treatment was significantly accelerated in comparison to those of the placebo-treated patients, indicating that the recovery of functional status of the injured hand was facilitated by LcS treatment.

As secondary outcomes of this study, active range of motion (ROM) measurements, including radial deviation and inclination, and ulnar deviation and variance, were also assessed. With the exception of ulnar variance, all other three parameters have exhibited more pronounced improvements in the LcS group than the placebo group, starting from as early as month 2 and lasted till the end of study. Even in the case of ulnar variance, a statistically significant improvement could be seen in the LcS group compared to that in the placebo group. These significantly improved ROM parameters among patients receiving LcS corresponded well with the above observed improved MHQ scores and further demonstrated the efficacy of oral LcS administration in accelerating hand function recovery.

It is also worthy of noting that the dose of LcS in our present study (1.2 × 10^10^ CFU) has been doubled compared to our previous study (6 × 10^9^ CFU), because we found that the positive effects of LcS on the functional recovery after fracture began to dissipate after the initial treatment [20]. One possibility is that these patients may have grown accustomed to the LcS treatment at lower dose; therefore, we raised the question whether a higher dosage of LcS could more effectively promote distal radius fracture recovery. Therefore, in this study, we increased the dose of LcS with the aim to confirm its efficacy as well as safety. Again, we did not observe any serious adverse events throughout the current study, nor did any patient report intolerance to the elevated dose of LcS. In previous reports regarding the clinical use of LcS in other diseases, study periods were normally 4 weeks [6–8] or even as short as 5 days [9]. Hence, our present study, with increased dose and lasted for 3 months, provides a significant assurance for not only the efficacy but also the safety of oral LcS administration in the clinic.

## Conclusion

In summary, we have discovered in the current clinical trial that a daily treatment of 1.2 × 10^10^ CFU LcS significantly enhanced the recovery of hand functions after distal radius fracture, starting from the second month post-injury. This finding provides important insights for patients who are suffering from acute distal radius facture, especially the elderly, because the recovery is most challenging right after the injury. The conclusion that an agent such as LcS could promote the initial healing would be not only a great relief to the patient, but also welcomed by families of the patients and the physicians.

## Data Availability

All data generated or analyzed during this study are included in this published article.

## Abbreviations

LcS: *Lactobacillus casei* Shirota
MHQ: Michigan Hand Questionnaire

## References

[1] Waljee Jennifer F., Ladd Amy, MacDermid Joy C., Rozental Tamara D., Wolfe Scott W., Benson Leon S., Calfee Ryan P., Dennison David G., Hanel Douglas P., Herzberg Guillaume, Hotchkiss Robert, Jupiter Jesse B., Kaufmann Robert A., Lee Steve K., Ozer Kagan, Ring David C., Ross Mark, Stern Peter J. (2016). A Unified Approach to Outcomes Assessment for Distal Radius Fractures. The Journal of Hand Surgery.

[2] Farr JN, Khosla S (2015). Skeletal changes through the lifespan--from growth to senescence. Nat Rev Endocrinol.

[3] Tulipan J, Jones CM, Ilyas AM (2015). The effect of osteoporosis on healing of distal radius fragility fractures. Orthop Clin North Am.

[4] Fuller R (1989). Probiotics in man and animals. J Appl Bacteriol.

[5] Salminen S, Ouwehand A, Benno Y, Lee YK (1999). Probiotics: how should they be defined?. Trends Food Sci Technol.

[6] Matsuzaki T, Saito M, Usuku K, Nose H, Izumo S, Arimura K (2005). A prospective uncontrolled trial of fermented milk drink containing viable Lactobacillus casei strain Shirota in the treatment of HTLV-1 associated myelopathy/tropical spastic paraparesis. J Neurol Sci.

[7] Stadlbauer V, Mookerjee RP, Hodges S, Wright GA, Davies NA, Jalan R (2008). Effect of probiotic treatment on deranged neutrophil function and cytokine responses in patients with compensated alcoholic cirrhosis. J Hepatol.

[8] Dong H, Rowland I, Thomas LV, Yaqoob P (2013). Immunomodulatory effects of a probiotic drink containing Lactobacillus casei Shirota in healthy older volunteers. Eur J Nutr.

[9] Srinivasan R, Meyer R, Padmanabhan R, Britto J (2006). Clinical safety of Lactobacillus casei Shirota as a probiotic in critically ill children. J Pediatr Gastroenterol Nutr.

[10] Gleeson M, Bishop NC, Oliveira M, Tauler P (2011). Daily probiotic’s (Lactobacillus casei Shirota) reduction of infection incidence in athletes. Int J Sport Nutr Exerc Metab.

[11] Quach D, Britton RA (2017). Gut microbiota and bone health. Adv Exp Med Biol.

[12] Schepper JD, Irwin R, Kang J, Dagenais K, Lemon T, Shinouskis A (2017). Probiotics in gut-bone signaling. Adv Exp Med Biol.

[13] Collins FL, Rios-Arce ND, Schepper JD, Parameswaran N, McCabe LR. The potential of probiotics as a therapy for osteoporosis. Microbiol Spectr. 2017;5(4). 10.1128/microbiolspec.BAD-0015-2016. 10.1128/microbiolspec.bad-0015-2016 PMC5710820 28840819

[14] Narva M, Halleen J, Vaananen K, Korpela R (2004). Effects of Lactobacillus helveticus fermented milk on bone cells in vitro. Life Sci.

[15] McCabe LR, Irwin R, Schaefer L, Britton RA (2013). Probiotic use decreases intestinal inflammation and increases bone density in healthy male but not female mice. J Cell Physiol.

[16] Britton RA, Irwin R, Quach D, Schaefer L, Zhang J, Lee T (2014). Probiotic L. reuteri treatment prevents bone loss in a menopausal ovariectomized mouse model. J Cell Physiol.

[17] Parvaneh K, Ebrahimi M, Sabran MR, Karimi G, Hwei AN, Abdul-Majeed S (2015). Probiotics (Bifidobacterium longum) increase bone mass density and upregulate Sparc and Bmp-2 genes in rats with bone loss resulting from ovariectomy. Biomed Res Int.

[18] Amdekar S, Singh V, Singh R, Sharma P, Keshav P, Kumar A (2011). Lactobacillus casei reduces the inflammatory joint damage associated with collagen-induced arthritis (CIA) by reducing the pro-inflammatory cytokines: Lactobacillus casei: COX-2 inhibitor. J Clin Immunol.

[19] Lee JM, Hwang KT, Jun WJ, Park CS, Lee MY (2008). Antiinflammatory effect of lactic acid bacteria: inhibition of cyclooxygenase-2 by suppressing nuclear factor-kappaB in Raw264.7 macrophage cells. J Microbiol Biotechnol.

[20] Lei M, Hua LM, Wang DW (2016). The effect of probiotic treatment on elderly patients with distal radius fracture: a prospective double-blind, placebo-controlled randomised clinical trial. Benef Microbes.

[21] Chung KC, Pillsbury MS, Walters MR, Hayward RA (1998). Reliability and validity testing of the Michigan Hand Outcomes Questionnaire. J Hand Surg Am.

[22] Cohen J (1992). Statistical power analysis. Curr Dir Psychol Sci.

[23] Gummesson C, Atroshi I, Ekdahl C (2003). The disabilities of the arm, shoulder and hand (DASH) outcome questionnaire: longitudinal construct validity and measuring self-rated health change after surgery. BMC Musculoskelet Disord.

[24] MacDermid JC, Richards RS, Donner A, Bellamy N, Roth JH (2000). Responsiveness of the short form-36, disability of the arm, shoulder, and hand questionnaire, patient-rated wrist evaluation, and physical impairment measurements in evaluating recovery after a distal radius fracture. J Hand Surg.

[25] Hudak PL, Amadio PC, Bombardier C (1996). Development of an upper extremity outcome measure: the DASH (disabilities of the arm, shoulder and hand) [corrected]. The Upper Extremity Collaborative Group (UECG). Am J Ind Med.

[26] Case-Smith J (2003). Outcomes in hand rehabilitation using occupational therapy services. Am J Occup Ther.

[27] Kotsis SV, Chung KC (2005). Responsiveness of the Michigan Hand Outcomes Questionnaire and the disabilities of the arm, shoulder and hand questionnaire in carpal tunnel surgery. J Hand Surg.

